# Freeze-dried Xanthan/Guar Gum Nasal Inserts for the Delivery of Metoclopramide Hydrochloride

**Published:** 2012

**Authors:** Mohamed Hassan Dehghan, Mohan Girase

**Affiliations:** *Department of Pharmaceutics, Dr Rafiq Zakaria Campus, Maulana Azad Educational Tust*’*s, Y. B Chavan College of Pharmacy, Aurangabad, Maharashtra, India.*

**Keywords:** Xanthan gum, Guar gum, Metoclopramide hydrochloride, Nasal Inserts, Bioadhesion, In-vitro drug release

## Abstract

Prolonged residence of drug formulation in the nasal cavity is important for the enhancing intranasal drug delivery. The objective of the present study was to develop a mucoadhesive *in-situ* gelling nasal insert which would enable the reduced nasal mucociliary clearance in order to improve the bioavailability of metoclopramide hydrochloride. Metoclopramide hydrochloride is a potent antiemetic and effective for preventing emesis induced by cancer chemotherapy, migraine, pregnancy and gastroparesis. It undergoes hepatic first pass metabolism and both the absolute bioavailability and the plasma concentrations are subjected to wide inter-individual variation showing values between 32% and 98%. Oral antiemetic often gets vomited out before the systemic absorption compelling parenteral administration which results in low patient compliance. Adverse effect of metoclopramide HCL on CNS caused by high plasma peaks can be avoided through sustained formulation.

A novel combination of xanthan gum and guar gum was used to prepare the nasal inserts and the effect of blend ratio of xanthan gum and guar gum on drug release from *in-situ* gelling nasal inserts and on other insert properties such as bioadhesion potential and water uptake was studied. PXRD was used to determine the effect of freeze-drying on crystalline nature of formulation.

The viscosities of xanthan gum in combination with guar gum were observed to be higher than that of single polymer solutions. This is because of the synergistic rheological interaction between xanthan and guar gum. There is a substantial loss in crystalline nature of the formulation after freeze-drying.

The best nasal inserts formulation containing xanthan gum and guar gum ratio 1:5, showed good release (91.83%) as well as bioadhesion which may result in an increase in the nasal residence time.

## Introduction

Intranasal administration represents a viable option for local and systemic delivery of diverse therapeutic compounds. The large surface area of the nasal mucosa affords a rapid onset of therapeutic effect, potential for direct-to-central nervous system delivery, no first pass metabolism, and non-invasiveness; all of which may maximize the patient’s convenience, comfort, and compliance (1). Nasal transmucosal medication delivery is a promising development of the pharmaceutical industry which may provide the clinicians using already available intravenous medications an alternative route. The nasal mucosa is an attractive area to which the medications are delivered since the procedure is painless and needleless, which eliminates the risk of needle-stick injuries and reduces the patient’s discomfort. Oral medications are about 5% to 10% less bioavailable owing to gastrointestinal and hepatic destruction (2). Mucoadhesion localizes the formulation within the nasal cavity for extended time period and increases the absorption which otherwise would not occur (3). The dual strategy of reducing the product’s development time by applying the existing excipients for nasal drug delivery has been successfully applied in the development of new products (4).

Metoclopramide hydrochloride is a potent antiemetic, effective in the treatment of nausea and vomiting associated with migraine, cancer therapy, pregnancy, *etc*. However, it undergoes hepatic first pass metabolism and both the absolute bioavailability and the plasma concentrations are subjected to wide inter-individual variation showing values between 32% and 98% (6, 7). Oral antiemetic drugs are often vomited out before the systemic absorption compelling parenteral administration which results in low patient compliance. If vomiting takes place, the amount of metoclopramide that remains in the stomach is unknown, and the result of treatment is even less predictable. Due to the nausea and vomiting associated with the gastroparesis, patients are even more reluctant to comply with the oral regimen.

Nasal insert is the novel solid dosage form, which is prepared by lyophilization, consists of a spongelike hydrophilic polymer matrix, in which the drug is embedded. It allows easy dosing with a high potential for systemic administration under the circumvention of the gastrointestinal tract-related harsh conditions and the hepatic first pass metabolism (5).

**Table 1:** Formulation of the nasal inserts of metoclopramide hydrochloride.

**Formulation Code**	**Xanthan: Guar Ratio**	**Xanthan gum (mg)**	**Guar gum (mg)**
**M1**	0:1	----	20
**M2**	1:0	20	----
**M3**	1:20	0.95	19.04
**M4**	1:10	1.81	18.17
**M5**	1:5	3.33	16.66
**M6**	1:3	5	15
**M7**	1:2	6.66	13.33
**M8**	2:1	13.33	6.66
**M9**	3:1	15	5
**M10**	1:1	10	10

Metoclopramide hydrochloride (10 mg) and mannitol (10 mg) were incorporated in each formulation; the total weight of polymer was maintained at 2% w/w of gel solution.

In the present work, nasal inserts containing generally regarded as safe (GRAS), excipients xanthan gum and guar gum innovel combinations were used to prepare themselves for the delivery of metoclopramide hydrochloride.

## Experimental

Metoclopramide hydrochloride was obtained as a gift sample from Cadila Pharmaceuticals Ltd. Ahmedabad, India. Xanthan gum was purchased from Research fine Lab. Mumbai, India. Guar gum was purchased from Loba Chem. Mumbai. Mannitol was procured from Merck (Merck, Mumbai). Cellulose acetate membrane (0.22 µm) was procured from Millipore, Bangalore, India. All other chemicals and reagents used were of Analytical Reagent Grade.

*Preparation of gel solutions*

Ten mg of drug and mannitol (1% w/w) was first dissolved in about one third of the required amount of distilled water. The required weight of xanthan gum and guar gum blend or individual polymer were then added slowly with constant stirring to the obtained uniform gels and the resulted solution was made up to the desired volume and stirred to homogeneity (Table 1), which was then stored at 4^°^C overnight to allow the removal of air bubbles.

*Preparation of nasal inserts*

**Figure 1:**
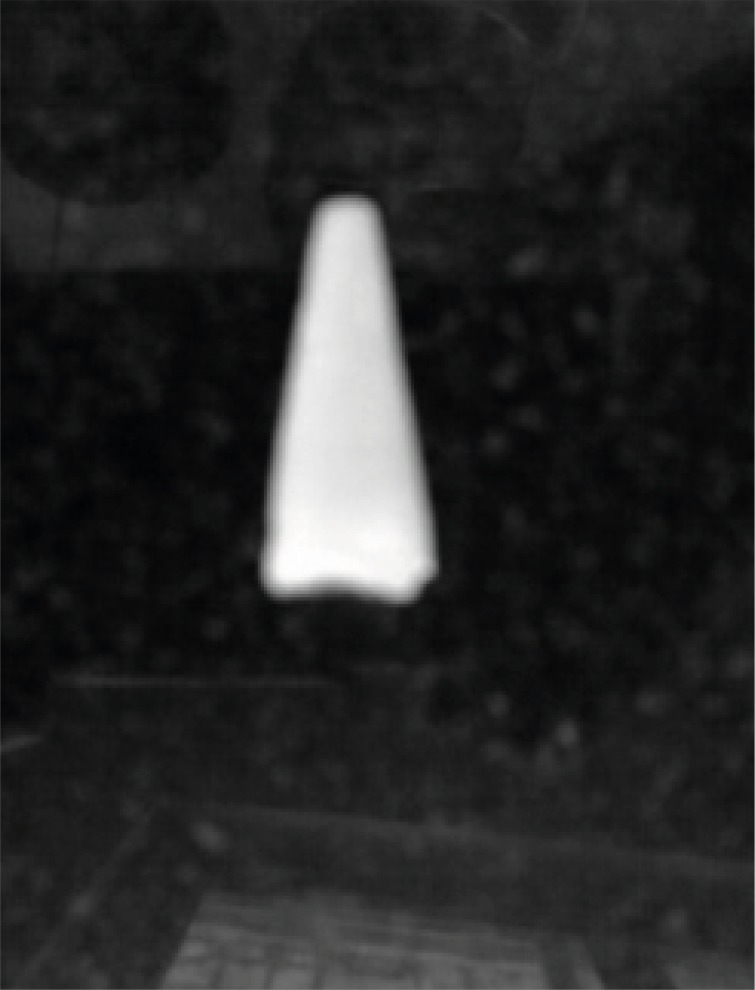
Shape of the freez dried nasal insert.

Aliquots of the prepared gel solution were filled into the polypropylene tubes (bullet shaped with internal diameter of 1 cm at the tube mouth and 3.5 cm in length) as moulds. The tubes were lyophilized for 24 h in a freeze-dryer with the pre-set cycle stages; freezed for 4 h, at -30°C, dried for 20 h, with vacuum 50 m Torr and condenser temperature at -50°C. The inserts were then stored in a desiccator until use (Figure 1).

*Viscosity measurement of polymer solutions*

The gel/sol samples were left undisturbed overnight and then equilibrated to 22°C ± 1°C for 1 h in a water bath before the measurement. The viscosity of the resultant polymer solutions were then determined with a Brookfield viscometerSpindle NO.64.

*In-vitro drug release*

A locally fabricated diffusion cell mimicking the humidity properties of the nasal mucosa, as reported by Roland Bodmeier (8), was used for the drug release studies. The lower end of a glass tube (inner diameter = 3.5 cm, surface area = 9.61 cm^2^) was closed with the cellulose acetate membrane (Millipore 0.22 µm pore size). This tube was placed vertically in a release medium container (filled with 50 mL phosphate buffer with pH of 6.0) and adjusted exactly to the height of the release medium surface so that the cellulose acetate membrane was wetted but not submersed. Briefly, the receptor compartment contained Phosphate buffer solution (PBS) with pH of 6 at 37°C and the donor compartment contained air saturated with moisture generated using the temperature and closed system nature of the experimental setup. The nasal insert was placed on the cellulose acetate membrane (Millipore 0.22 µm pore size) maintained just in contact with the liquid phase of the receptor compartment, which was constantly agitated with a Teflon coated magnetic bead. Samples of 1 mL were withdrawn at the regular time intervals from the receptor compartment and analyzed spectrophotometrically (using a UV double beam spectrophotometer) at 309 nm. Each sample taken from the receptor compartment was replaced immediately with 1 mL of fresh medium.

*Water uptake*

A sponge (5 cm × 6.5 cm × 3 cm) was fully soaked in the hydration medium (phosphate buffer with pH of 6.0) and placed in a petri dish filled with the same buffer to a height of 1 cm in order to keep the sponge soaked during the experiment. Circular filter paper (d = 55 mm, Whatman No.41) was also soaked in the medium and positioned on the top of sponge. This experimental setup was equilibrated for 30 min. Accurately weighed inserts were then placed on the filter paper and the water uptake was determined as the increase in the weight of insert (weight of hydrated insert and wet filter paper minus weight of wet filter paper) over the initial dry insert weight.

**Figure 2:**
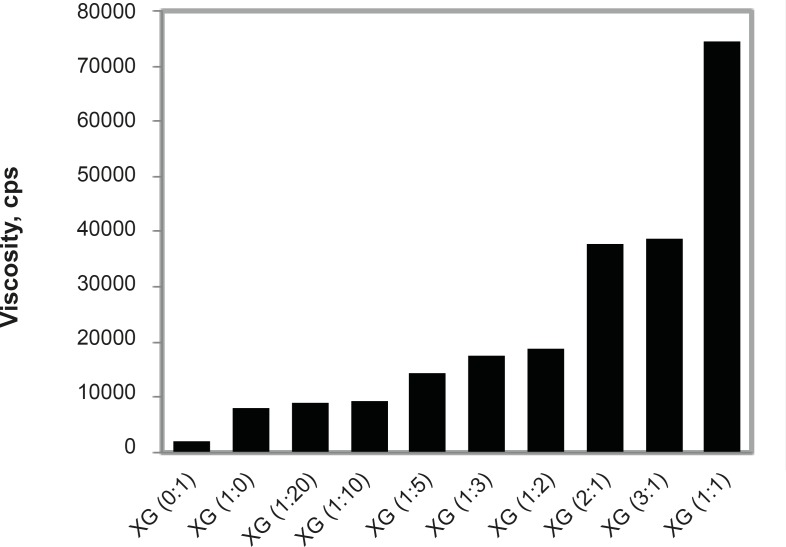
Viscosity synergism between Xanthan gum and Guar gum.

*Bioadhesive potential of inserts*

A hundred g of a hot agar solution (1%w/w, in phosphate buffer with pH of 6.0) was casted on a petri plate and left to gel at 4-8°C in a refrigerator for 3 h. The agar gel was then equilibrated for 1 h to the test conditions of 22°C and 79% relative humidity (saturated ammonium chloride solution) in a chamber. The inserts which were placed on the top of the agar gel, moved downward due to the gravity after the glass plate was turned into a vertical position. The displacement in mm was measured as a function of time (n = 3). The adhesive potential was inversely related to the displacement of the insert.

*Powder X-ray diffraction (PXRD)*

PXRD patterns were studied in order to evaluate the crystalline/amorphous character of untreated drug and inserts prepared by the freeze-drying of polymer solutions (2%, w/w). Measurements were performed using a Philips X-ray generator PW 1830 equipped with a copper anode (30 mA, 40 Kv) coupled to a computer-interfaced diffractometer control unit (XPERT-PRO). The scattered radiation was measured with a vertical goniometer (PW3050/60).

## Results and Discussion

*Viscosity measurement of polymer solutions*

The *in-situ* gelling nasal inserts take fluid from the nasal mucosa and form a gel. The viscosity of this gel or that of the polymer solution for insert preparation is of utmost importance for the performance of inserts with respect to drug release, water uptake, as well as bioadhesion. The viscosities of polymer solutions (2% w/w) were measured using Brookfield viscometer. Xanthan gum (2% w/w) shows higher viscosity than guar gum (2% w/w). This finding corroborates with that of J. L. Amundarain *et al.* (8). Viscosities of polymer blends of xanthan and guar gum with varying ratios were measured. The viscosities of xanthan gum in combination with guar gum were observed to be higher than that of single polymer solutions. This is because of the synergistic rheological interaction between xanthan and guar gum. The synergy is thought to be a consequence of direct interaction between the xanthan gum chains and exposed mannose segments in the backbone of the guar gum macromolecule. The ability of the xanthan and guar gum molecules to interact is responsible for the synergistic effect. The interaction leads to cooperative structures that enhance the existence of entangled networks of polymer molecules, leading to the increased friction and higher viscosities. As the fraction of xanthan gum in polymer blend increases, viscosity goes on increasing. Maximum synergy and therefore viscosity was found for the Xanthan: Guar ratio (1:1) (Figure 2).

**Figure 3:**
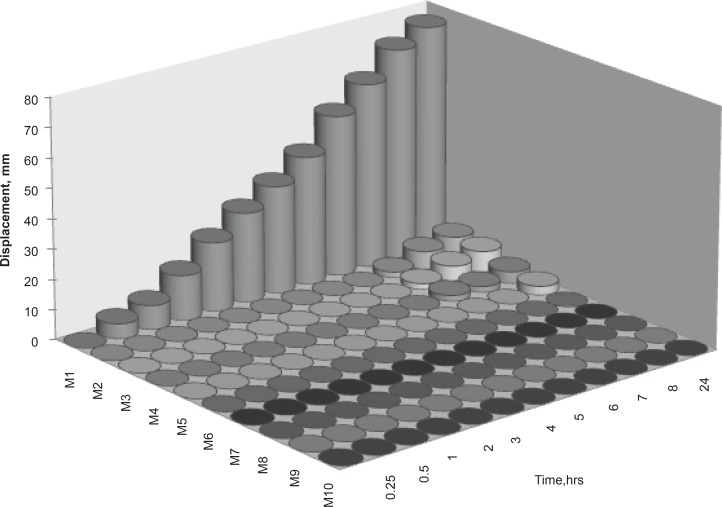
Bioadhesive profiles of nasal insert formulations (2% polymer concentration, n = 3).

*In-vitro drug release*

**Figure 4:**
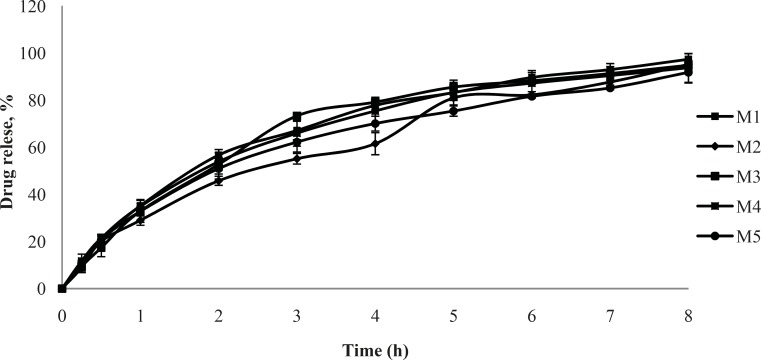
Cumulative % drug release vs. time profile for formulation M1, M2, M3, M4, M5 (2% polymer concentration, n = 3).

**Figure 5:**
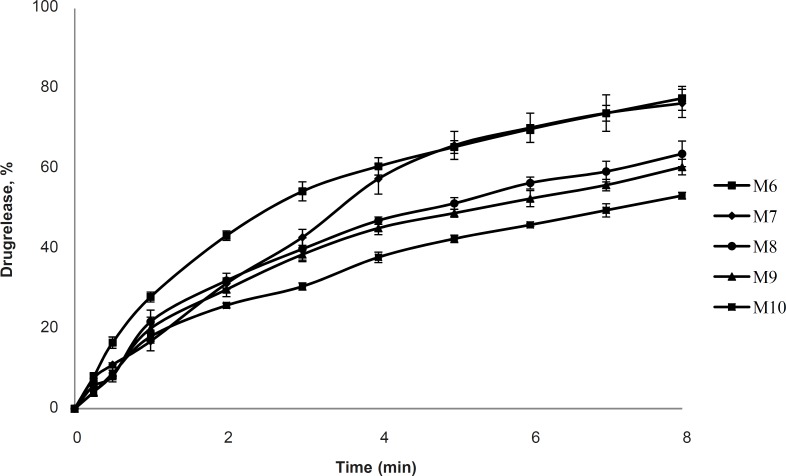
Cumulative % drug release vs. time profile for formulation M6, M7, M8, M9, M10 (2% polymer concentration, n = 3).

The cumulative % drug release versus time profile for the nasal inserts is given in Figures 4 and 5. Faster drug release in M1 formulations may be due to the lower viscosity in comparison with M2 formulation. There exists an inverse relationship between the viscosity and drug release, the apparent viscosity or microviscosity of the formulation which influences the diffusion of the particles, when the characteristic length is larger than the length scale of the structure elements in the formulation. As reported by Krishnaiah Y.S.R. (9), initial swelling of the guar gum may aid the dissolution of the freely soluble drugs and the dissolved drug diffuses out of the swollen gel barrier into the dissolution medium. The matrices made with xanthan gum alone showed higher drug retention, compared with the guar gum matrices. These findings are supported by Patel V. F. and Patel N. M. (10) who reported that the overall rate of drug release from guar gum is higher than that of xanthan gum matrices; it clearly indicates that xanthan gum has higher drug retarding ability than guar gum. Similarly, in another study of xanthan and galactomannan (from *M. scabrella*) matrix tablets for oral controlled delivery of theophylline (11), it was found that the drug release was slower than the matrices with xanthan alone (8-25%) compared to the xanthan : guar matrices for the same total polymer concentration. These results point out the major role of xanthan gum in drug release of matrices containing xanthan : guar mixtures. Drug release may effectively be controlled by varying the polymer blend ratio. Further drug release from hydrophilic swellable matrices is controlled via diffusion through the gel layer for water-soluble drugs. In earlier studies, xanthan and galactomannan matrices have shown constant drug release profile with combination of diffusion and relaxation (10).

*Water uptake*

**Figure 6:**
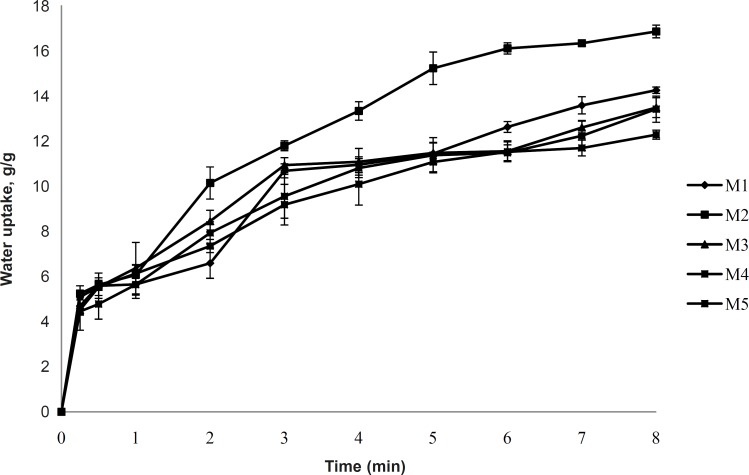
Water uptake study of formulations M1-M5 (2% polymer concentration, n = 3).

**Figure 7:**
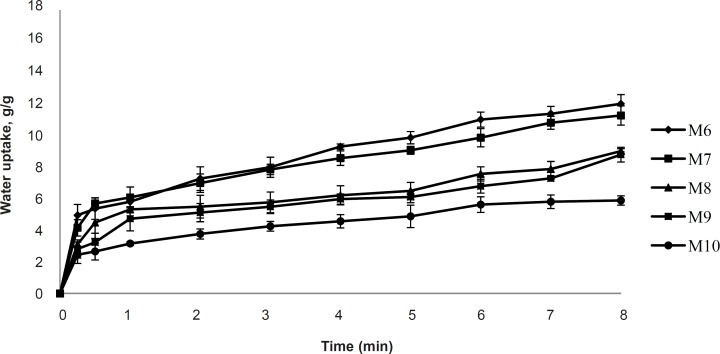
Water uptake study of formulations M6-M10 (2% polymer concentration, n = 3).

The uptake of water by *in-situ* gelling inserts is a crucial step for the transformation into gel and for adhesion to the mucosa. Formulation M2 shows the highest water uptake as shown in Figure 6. It may be attributed to the high charge density of xanthan gum. This finding corroborated with Mundargi R. C and Patil S. A (12) that the formulation containing xanthan gum showed the highest swelling ratio among the formulations containing xanthan gum, mixture of xanthan with Methacrylic Acid-g-GG or GG with Methacrylic Acid-g-GG. There is a strong degree of water uptake by xanthan gum (12). Hydration of xanthan gum occurs when hydrogen bonding forces maintain the integrity of the hydrophilic gum matrix (13, 14). M1 shows lower water uptake than M2 because of the non-ionic nature of the guar gum, whereas, the formulations of M3-M10 show decreases in water uptake (Figures 6 and 7). Inserts from blends of xanthan gum and guar gum showed a reduced water uptake compared to the pure xanthan and pure guar gum. Reduced viscosity of the hydrating inserts and reduces the diffusion barrier function of gel matrix for the water influx.

*Bioadhesion potential of inserts*

The vertical displacement of inserts on an agar gel was used as a measurement of bioadhesive potential. An almost instantaneous displacement and thus low bioadhesion was observed in formulation M1 containing only guar gum. Negligible displacement was observed for xanthan gum inserts (M2). Formulations M3-M10 showed very little or even no displacement and thus, are highly bioadhesive compared to M1 and M2. Higher bioadhesion of xanthan gum may be due to the polymer possessing of a negative charge and high solution viscosity. The positive bioadhesion performance of negatively charged *in-vivo* polymers has been reported to be related to their good balance between the available hydrogen bonding sites and an open expanded conformation (15). Guar gum shows an inability to interact with mucin either electrostatically or by entanglements due to the low solution viscosity or its neutral nature.

*Powder X-ray diffraction (PXRD)*

**Figure 8:**
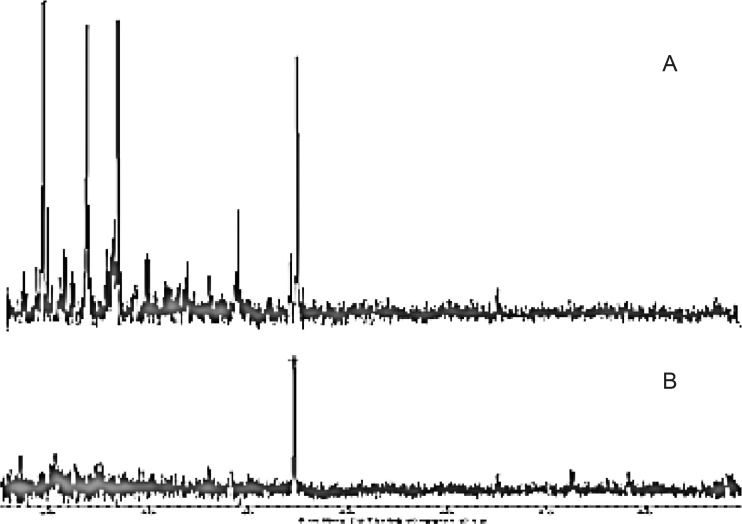
PXRD pattern of (A) Physical mixture and (B) Freeze-dried formulation.

The diffraction pattern of physical mixture was highly crystalline in nature as indicated through numerous peaks. Physical mixture shows sharp peaks 2 values 18.17, 23.31, 25.97, 26.44, 29.43, 38.62, 44.64 were noticeable and indicating crystalline nature of drug. The maximum intensity peak was found to at peaks 2 values equal to 44.64. In case of diffractogram, the freeze-dried formulation showed large reduction in peas’ characteristics, whereas, peaks at peaks 2 values equal to 44.65 with much reduced peak height from 119.98 (physical mixture) to 66.99 (freeze-dried). Moreover, a large reduction in the peaks characteristics indicated a substantial decrease in crystalline nature of the mixture in freeze-dried formulation (Figure 8).

## Conclusion

The nasal insert prepared by freeze-drying method containing blends of the xanthan and guar gum retards metoclopramide hydrochloride release as compared to the insert containing xanthan and guar gum alone. The use of polymer blends presented broad range of drug release rates, whereas the nasal inserts from the blends of xanthan gum and guar gum showed excellent bioadhesive potential when compared with inserts of xanthan and guar alone. There is substantial loss in crystalline nature of the formulation after the freeze-drying.

Finally, it can be concluded that *in-situ *gelling bioadhesive nasal inserts were successfully designed for prolonged delivery of metoclopramide hydrochloride. The best nasal inserts formulation containing xanthan gum : guar gum (1 : 5), showed good release (91.83%) as well as bioadhesion which may result in an increase in the nasal residence time.
